# Heterogeneous natural history of Addison’s disease: mineralocorticoid deficiency may predominate

**DOI:** 10.1530/EC-22-0305

**Published:** 2022-12-15

**Authors:** Sophie Howarth, Luca Giovanelli, Catherine Napier, Simon H Pearce

**Affiliations:** 1 Clinical and Translational Research Institute, Newcastle University, Newcastle upon Tyne, UK; 2 Department of Endocrinology, The Newcastle upon Tyne Hospitals NHS Foundation Trust, Newcastle upon Tyne, UK

**Keywords:** Addison’s disease, autoimmunity, adrenal insufficiency, mineralocorticoid, glucocorticoid, cortisol

## Abstract

Autoimmune Addison’s disease (AAD) is defined as primary adrenal insufficiency due to immune-mediated destruction of the adrenal cortex. This destruction of steroid-producing cells has historically been thought of as an irreversible process, with linear progression from an ACTH-driven compensated phase to overt adrenal insufficiency requiring lifelong glucocorticoid replacement. However, a growing body of evidence suggests that this process may be more heterogeneous than previously thought, with potential for complete or partial recovery of glucocorticoid secretion. Although patients with persistent mineralocorticoid deficiency despite preserved or recovered glucocorticoid function are anecdotally mentioned, few well-documented cases have been reported to date. We present three patients in the United Kingdom who further challenge the long-standing hypothesis that AAD is a progressive, irreversible disease process. We describe one patient with a 4-year history of mineralocorticoid-only Addison’s disease, a patient with spontaneous recovery of adrenal function and one patient with clinical features of adrenal insufficiency despite significant residual cortisol function. All three patients show varying degrees of mineralocorticoid deficiency, suggesting that recovery of zona fasciculata function in the adrenal cortex may occur independently to that of the zona glomerulosa. We outline the current evidence for heterogeneity in the natural history of AAD and discuss possible mechanisms for the recovery of adrenal function.

## Introduction

Autoimmune Addison’s disease (AAD) is a rare endocrinopathy with a prevalence of 110–140 cases per million in western populations (1). AAD can occur in isolation but is frequently seen alongside other autoimmune conditions such as type 1 diabetes, autoimmune hypothyroidism and pernicious anaemia.

Complex interplay between genetic and environmental factors gives rise to immune-mediated destruction of the steroid-producing cells of the adrenal cortex. As in type 1 diabetes, this immune process is felt to be primarily T-cell mediated, with previous work demonstrating T cells specific for the steroidogenic enzyme 21-hydroxylase (2). Autoantibodies against 21-hydroxylase are positive in >85% of patients, becoming detectable prior to the onset of symptoms and persisting for several decades (3).

With the exception of autoimmune polyglandular syndrome 1, which is associated with consistent rapid progression to adrenal insufficiency in childhood, our understanding of the natural history of AAD has mirrored that seen in well-characterised conditions such as type 1 diabetes. AAD is felt to have a preclinical phase, where 21-hydroxylase antibodies are detectable, but the immune-mediated destruction has not become clinically or biochemically manifest (4). A proportion (~50%) of these patients progress to adrenal cortical destruction (5) but are able to compensate by adrenocorticotrophic hormone (ACTH)-driven hypertrophy of unaffected areas, as demonstrated by case reports of post-mortem adrenal gland pathology showing areas of adrenal gland hypertrophy alongside fibrosis (6). This compensated phase with gradual skin hyperpigmentation is then followed by a symptomatic phase, where adrenal steroid secretion becomes insufficient to meet the body’s needs. Overt mineralocorticoid deficiency causes the characteristic presentation of salt craving, postural hypotension, hyponatraemia and hyperkalaemia. Glucocorticoid deficiency causes loss of appetite, early satiety, weight loss, hypoglycaemia and fatigue.

Historically, the symptomatic phase was felt to be a point of no return for Addison’s patients, with a lifelong requirement for steroid replacement to avoid fatal adrenal crisis. However, a growing body of evidence suggests that the natural history of AAD is, in fact, non-linear and may be more heterogeneous than previously thought. Two cases of spontaneous adrenal function recovery in antibody-positive patients are documented in the literature, though one of these patients required continued mineralocorticoid replacement (7, 8). An additional third case of complete resolution did not have confirmation of autoimmune aetiology (9). Furthermore, several studies have documented the presence of residual glucocorticoid secretion in a sizeable minority of AAD patients, and this has formed the rationale for preliminary clinical trials investigating the use of immunosuppressive treatment or tetracosactide (synacthen, ACTH_1-24_) to promote adrenal recovery (10, 11, 12, 13).

We present three cases that typify the heterogeneous nature of AAD, including two cases where mineralocorticoid deficiency dominated the clinical picture for many years and one case of spontaneous remission. We discuss the possible physiological mechanisms underlying the different presentations and the possible implications for future research.

### Inclusion criteria

All three patients had presented to the adrenal clinic at the Newcastle upon Tyne Hospitals NHS Foundation Trust for routine clinical care within the last two decades. The adrenal clinic follows approximately 100 patients with AAD. Patients were included in the cases series if they had prolonged evidence of preserved glucocorticoid secretion with biochemical or clinical evidence of mineralocorticoid deficiency. This was defined as a normal short synacthen test (250µg ACTH_1-24_), with either a low aldosterone and raised plasma renin or clinical need for fludrocortisone supplementation.

## Patient 1: mineralocorticoid-only Addison’s disease

A 36-year-old woman with a background of type 1 diabetes, pernicious anaemia, autoimmune hypothyroidism and obesity was noted to have persistent hyponatraemia (Na^+^ 132 mmol/L) and hyperkalaemia (K^+^ 6.0 mmol/L). Her medication history included levothyroxine 200 µg once daily, 3-monthly vitamin B12 injections, an actrapid/glargine basal bolus insulin regimen and the combined oral contraceptive pill (COCP). Eight years previously, she had been hospitalised for an episode of gastroenteritis which was complicated by hyponatraemia (Na 129 mmol/L).

She reported postural dizziness after bathing, leg cramps, one to two episodes of nocturia nightly and salt craving. Importantly, she did not report any weight loss and her blood glucose was stable. Her plasma renin was found to be elevated at >500 mIU/L (mass assay, reference range 5–99), with an aldosterone of <103 pmol/L and HbA1c 73 mmol/mol. Urine albumin–creatinine ratio was normal (0.6 mg/mmol). There was no postural hypotension in clinic, but, given the strong biochemical evidence of mineralocorticoid deficiency, she was started on fludrocortisone 100 µg once daily and provided with an emergency supply of hydrocortisone to use in the event of illness.

Subsequent investigation showed normalisation of her serum electrolytes with a persistently elevated renin (372.8 mIU/L) during fludrocortisone treatment. Following COCP withdrawal, she had a basal cortisol of 344 nmol/L, rising to 452 nmol/L 60 min post-injection of 250 µg synacthen (ACTH_1-24_; reference >420nmol/L), showing preserved glucocorticoid secretion. ACTH was raised at 83 ng/L (reference 7.2–63.3 ng/L). 21-Hydroxylase antibodies were positive (39.9 U/mL; reference <1.0).

Over the course of 4 years, her adrenal insufficiency remained well-controlled on fludrocortisone alone, taking prophylactic oral hydrocortisone once when she got a tattoo and once for migraine-induced vomiting. Four years after diagnosis, her peak cortisol on short synacthen test fell below the reference range (0 min = 309 nmol/L, 30 min = 323 nmol/L, 60 min = 344 nmol/L, on a progesterone only contraceptive pill), and hydrocortisone 15 mg daily in a split dose was started (Table 1). However, she quickly developed ankle swelling which resolved after reducing the hydrocortisone dose to 5 mg once daily. At the time of writing, she remains well on hydrocortisone 5 mg mane and fludrocortisone 100 µg once daily.

**Table 1:** ACTH and cortisol values displayed at presentation and after the follow-up period showing preservation of glucocorticoid secretion in all three patients.

	Patient 1	Patient 2	Patient 3
**At presentation**			
ACTH (ng/L)	83	332	188
Basal cortisol (nmol/L)	344	421	360
Peak cortisol post synacthen (nmol/L)	452	389	430
Follow up: time (years)	4	18	5
ACTH (ng/L)	91	101	126
Basal cortisol (nmol/L)	309	326	442
Peak cortisol post synacthen (nmol/L)	344	529	458

## Patient 2: spontaneous resolution of Addison’s disease

A 51-year-old man undergoing investigation for a choreiform movement disorder was found to have persistent hyponatraemia (serum Na 129 mmol/L), 13 kg weight loss and fatigue. He had no personal or family history of autoimmune disease and his comorbidities included childhood poliovirus infection and COPD, for which he did not require any medication. On clinical examination, there was buccal and skin-fold pigmentation. Peak cortisol following synacthen was 250 µg, which was below the reference range (389 nmol/L), ACTH was raised at 332 ng/L and adrenocortical cell antibodies were positive by direct immunofluorescence. CT scan of his abdomen and pelvis showed normal adrenal gland appearances. He was treated as AAD with fludrocortisone 100 µg once daily and hydrocortisone 20 mg/24 h (10 mg in the morning, 5 mg at lunchtime and 5 mg in the afternoon). His serum sodium normalised and objective cognitive improvement was reported by the neurology team.

Over the course of 10 years, he remained on hydrocortisone and fludrocortisone with no adrenal crises. Eleven years post-diagnosis, his 17-alphahydroxyprogesterone was found to be detectable during biochemical workup for a clinical trial. Repeat short synacthen test off hydrocortisone confirmed adequate glucocorticoid secretion (0 min = 376 nmol/L, 30 min = 687 nmol/L, 60 min = 727 nmol/L), and his glucocorticoid and fludrocortisone were stopped. Following the cessation of his hydrocortisone, he developed arthralgia and worsening shortness of breath. Rheumatological investigations were negative, and his respiratory symptoms improved with fluticasone/salmeterol inhaler.

Seven years after stopping his hydrocortisone and fludrocortisone, he remains well with no clinical symptoms of adrenal insufficiency except intermittent mild salt cravings. His 21-hydroxylase antibodies remain positive (2.5 U/mL; reference <1.0) and his ACTH and plasma renin remain elevated at 91 ng/L and 171.9 mIU/L, respectively. He keeps an emergency supply of hydrocortisone at home for the event of illness and remains under annual adrenal clinical follow-up.

## Patient 3: preserved glucocorticoid secretion with symptomatic mineralocorticoid deficiency

A 20-year-old woman presented with 10 kg weight loss, fatigue, salt craving and hyperpigmentation. She had a past medical history of autoimmune hypothyroidism (TSH receptor and thyroglobulin antibody positive), premature ovarian insufficiency and migraine. Synacthen testing revealed preserved glucocorticoid secretion (0 min = 360 nmol/L, 30 min = 430 nmol/L) with a raised ACTH of 188 ng/L. Given her prominent symptoms including weight loss, she was started on hydrocortisone 15 mg/5 mg and fludrocortisone 150 µg once daily.

Her symptoms improved with steroid replacement, but she reported intermittent postural hypotension following night-time shift work which required additional stress hydrocortisone dosing. Five years after diagnosis, her synacthen test was repeated off steroids as preparation for a clinical trial and showed preserved glucocorticoid function (0 min = 442 nmol/L, 30 min = 465 nmol/L, 60 min = 458 nmol/L) with an elevated ACTH of 126 ng/L. At the time of writing, she remains well on hydrocortisone 10mg/5mg, fludrocortisone 150 µg and levothyroxine 112.5 µg daily.

## Discussion

In this case series, we present three patients with partial adrenal insufficiency owing to AAD. All three exhibited preserved cortisol secretion and varying degrees of mineralocorticoid deficiency ranging from intermittent mild salt craving to characteristic adrenal crisis symptoms after periods of stress. Although isolated aldosterone deficiency has been anecdotally mentioned by clinicians, there are few reports of such patients in the literature (14, 15).

Following the detection of adrenal antibodies, it is known that the onset of adrenal insufficiency in AAD may be slow. Raised ACTH may well be the first biochemical abnormality detected (16), and one case report documented a 9-year interval between the onset of ACTH-driven hyperpigmentation and the clinical diagnosis of AAD (17). It is logical that the immune-mediated destruction can begin in one part of the adrenal gland (indeed potentially in only one gland), leaving other parts of the adrenal cortex able to undergo compensatory hypertrophy in response to elevated plasma ACTH. All patients in our case series showed persistent compensatory ACTH increase; however, case 1 was notable for her reduction in cortisol secretion on repeat testing 4 years post-diagnosis. This finding in case 1 may represent slow progression of disease, with initial mineralocorticoid deficiency predominating for several years while glucocorticoid deficiency slowly develops. It has been hypothesised that the high concentrations of cortisol in the zona fasciculata (zF) (10–50 µM) may be sufficient to cause anergy of antigen-presenting cells and offer a degree of protection from immune attack (18, 19, 20). The mineralocorticoid-producing cells of the zona glomerulosa (zG) may be more vulnerable to immune-mediated destruction, giving rise to mineralocorticoid deficiency without glucocorticoid deficiency. The second and third cases in this series demonstrate that progression to permanent glucocorticoid deficiency is not inevitable in these patients.

All of our cases had significant residual endogenous cortisol secretion, allowing either a reduced dose of hydrocortisone to be given or none at all. It is important for clinicians to be aware of the heterogeneity in endogenous glucocorticoid secretion to prevent over-replacement with potential deleterious effect on bone density and metabolic profile. Case 2 in our series is the third documented case of antibody-positive Addison’s disease to go into spontaneous recovery after several years of glucocorticoid deficiency. Part of this phenomenon could be underpinned by ACTH-driven hypertrophy of the remaining differentiated cortical adrenocytes, but significant attention has also been given to the hypothesis of adrenal regeneration from adrenocortical stem cells (ACSCs). Rodent model studies have shown that ACSCs are present in the subcapsular region of the adrenal cortex and migrate centripetally, differentiating into zG cells and then zF cells under the influence of ACTH (21, 22). However, this does not explain why a degree of mineralocorticoid deficiency seems to remain in humans, with our patient reporting intermittent salt-craving symptoms and one of the two cases in the literature requiring ongoing fludrocortisone supplementation. It is a possibility that some glucocorticoid-secreting cells in the human zF are derived directly from ACSCs, bypassing the zG. Indeed, Freedman *et al.* demonstrated in the mouse that centripetal lineage conversion of zG cells into zF cells was the main pathway during postnatal adrenocortical development but that an alternative pathway for the regeneration of zF cells also existed. In zG-specific steroidogenic factor 1 knockout studies, where the zG was dedifferentiated and unable to contribute to the zF, they showed that the zF functioned normally and had an alternative cellular origin (23). This alternative regenerative pathway could account for the preserved zF function in the presence of persisting ‘zG deficiency’ in our patients.

This growing body of evidence gives hope that, for a minority of patients, residual adrenal function may be recovered or enhanced to reduce reliance on medication and the occurrence of life-threatening adrenal crises. Cross-sectional studies from the UK and Scandinavia found that the proportion of patients with residual cortisol secretion on synacthen testing was 15–30%, occurring more commonly in men and in those with a shorter duration of disease (24, 25). However, the presence of detectable cortisol secretion was not associated with reduced incidence of adrenal crises or improved quality of life. Peak serum cortisol correlated with plasma ACTH, supporting the hypothesis that ACTH-driven hypertrophy and regeneration underpins residual adrenal function (24). Small, preliminary trials into the use of tetracosactide (ACTH) and rituximab to promote adrenal function recovery have shown mixed results, with a handful of patients showing sustained steroid independence (10, 11, 12). These results may even underestimate the potential for adrenal recovery, as the patients in all three trials were taking exogenous steroids during the study period. It is plausible that exogenous steroid replacement could compound immune attack of the adrenal glands by suppressing the ACTH drive, reducing endogenous adrenal steroidogenesis and thus lowering the concentration of cortisol in the zF. Though the participants were recruited within 4 weeks of diagnosis, one study demonstrated a >50% reduction in endogenous steroid production in the 4 weeks after starting glucocorticoid replacement (25). The nature of Addison’s disease means that endogenous steroid replacement is likely to remain a complicating factor for future studies.

In summary, we present three case histories which outline the heterogeneous natural history of AAD. These studies highlight the need for clinicians to be aware of the possibility of residual adrenal function to avoid unnecessary glucocorticoid supplementation with potential side effects.

## Declaration of interest

SHP has consulted for Apitope/Worg and received speaker fees from Merck and IBSA. All other authors have no conflict of interest to declare.

## Funding

This work did not receive any specific grant from any funding agency in the public, commercial, or not-for-profit sector.

## Ethical approval

All procedures were carried out as part of routine NHS clinical care, and ethics committee review is not required.

## Patient consent

Informed consent was received from all patients involved in this case series.
